# 133. A Review of Antimicrobial Formularies at Rural Hospitals: Stewardship Opportunities Abound

**DOI:** 10.1093/ofid/ofab466.335

**Published:** 2021-12-04

**Authors:** Peter Bulger, Alyssa Y Castillo, John B Lynch, John B Lynch, Paul Pottinger, Jeannie D Chan, Rupali Jain, Mandana Naderi, Zahra Kassamali, Chloe Bryson-Cahn

**Affiliations:** 1 University of Washington, Seattle, Washington; 2 UW Medicine, Harborview Medical Center, Seattle, WA; 3 University of Washington School of Medicine, Seattle, WA; 4 University of Arizona College of Pharmacy, Phoenix, Arizona; 5 UW Medicine, Valley Medical Center, University of Washington, Seattle, WA

## Abstract

**Background:**

Management of a hospital’s antimicrobial formulary is an important aspect of antimicrobial stewardship and cost containment strategies. Ensuring that essential medications for clinical care are available and excluding therapeutic duplicates and unnecessary antimicrobials is time and resource intensive. Comparisons of antimicrobial formularies across multiple rural hospitals have not been evaluated in the literature. We hypothesized that a comprehensive formulary evaluation would reveal important opportunities for antimicrobial stewardship efforts and could help smaller hospitals optimize available medications.

**Methods:**

The University of Washington Tele-Antimicrobial Stewardship Program (UW-TASP) is comprised of 68 hospitals of varying sizes, most of which are rural and critical access, in Washington, Oregon, Arizona, Idaho, and Utah. We surveyed UW-TASP participating hospitals and other networked rural hospitals in multiple Western states using REDCap, a HIPAA-compliant, electronic data management program. Respondents reported which antimicrobials are on their hospital formulary as well as basic information about hospital size and inpatient units. Data were reviewed by a panel of infectious diseases trained physicians and pharmacists at UW-TASP.

**Results:**

Surveys from 49 hospitals were received; two were excluded from the data analysis (Table 1) – one submission was incomplete, and one was a large inpatient psychiatric hospital. Select antimicrobials and proportion of hospitals carrying these agents is shown in Table 2. Several antimicrobials are on the formulary at all hospitals, regardless of size. In some critical access hospitals (< 25 beds), empiric first-line bacterial meningitis and viral encephalitis coverage (Table 3) was lacking. Six hospitals (12.7%) lacked ampicillin for *Listeria* coverage and only one had a suitable alternative agent (meropenem). Seven hospitals (14.9%) lacked intravenous acyclovir, although three had oral valacyclovir. Formulary inclusion of agents for multi-drug resistant organisms was rare.

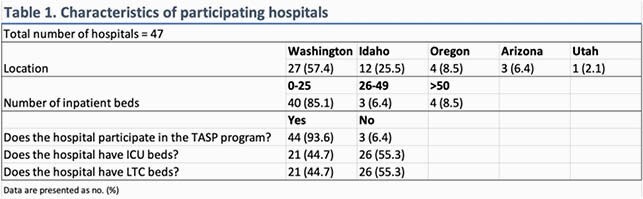

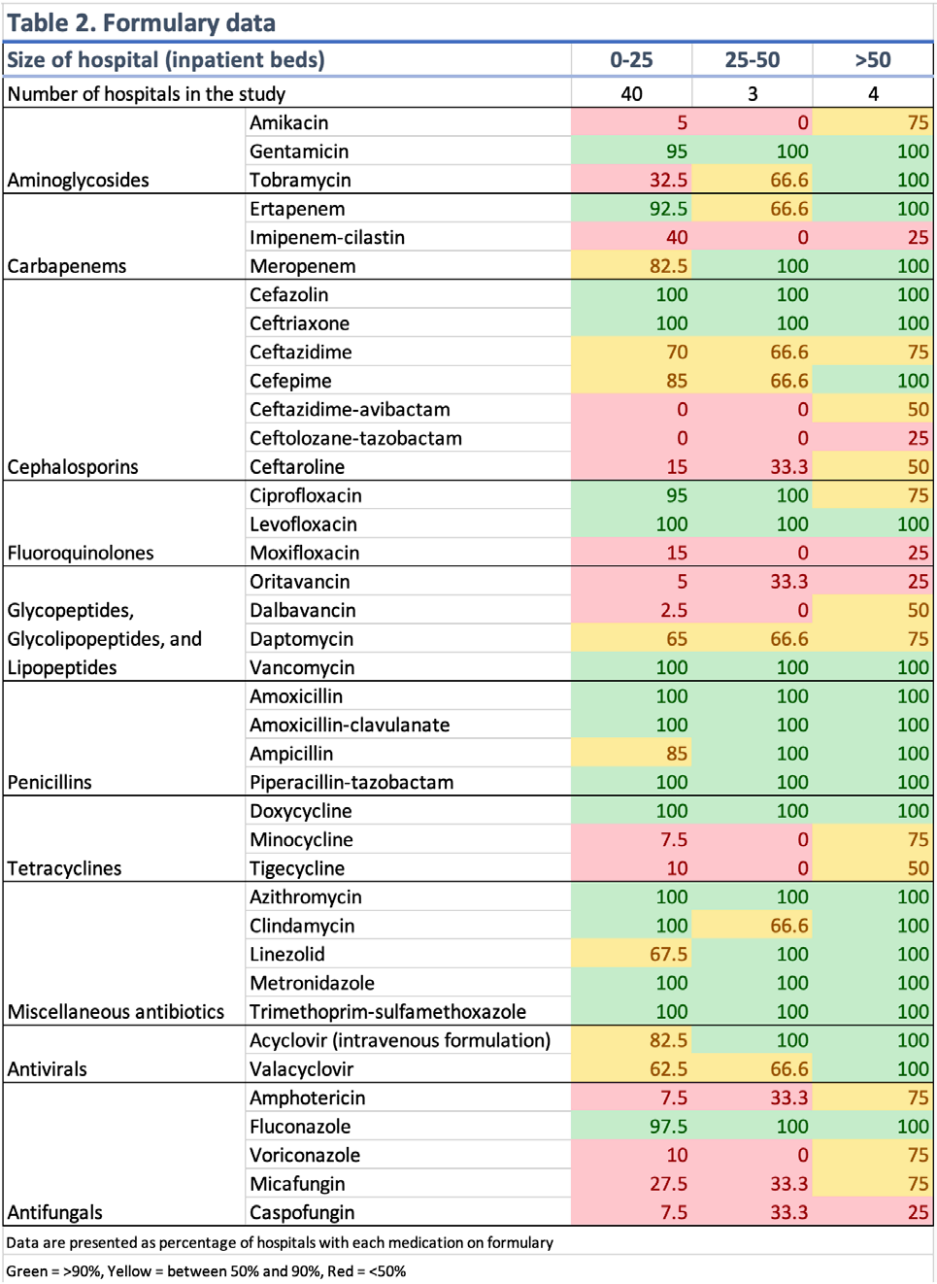

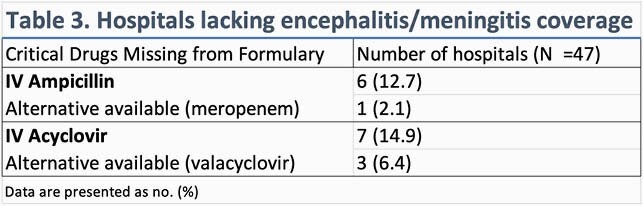

**Conclusion:**

In critical access hospitals in the Western USA, lack of essential empiric antimicrobials may be more of a concern than inclusion of agents with unnecessarily broad spectra.

**Disclosures:**

**Chloe Bryson-Cahn, MD**, **Alaska Airlines** (Other Financial or Material Support, Co-Medical Director, position is through the University of Washington)

